# Together We Stand, Divided We Fall: A Multidisciplinary Approach in Complicated Acute Pancreatitis

**DOI:** 10.3390/jcm8101607

**Published:** 2019-10-03

**Authors:** Jorge Paulino, Gonçalo Ramos, Filipe Veloso Gomes

**Affiliations:** 1 Department of Surgery, Centro Hepatobiliopancreático e Transplantação, Centro Hospitalar Universitário de Lisboa Central, Hospital Curry Cabral, Universidade Nova de Lisboa, 1050-099 Lisboa, Portugal; 2 Department of Gastroenterology, Centro Hospitalar Universitário de Lisboa Central, Hospital dos Capuchos, Universidade Nova de Lisboa, 1169-050 Lisboa, Portugal; goncalo.o.ramos@gmail.com; 3 Interventional Radiology Unit, Centro Hepatobiliopancreático e Transplantação, Centro Hospitalar Universitário de Lisboa Central, Hospital Curry Cabral, Universidade Nova de Lisboa, 1050-099 Lisboa, Portugal; fvgomes@gmail.com

**Keywords:** acute pancreatitis, infection, haemorrhage, necrosectomy, drainage

## Abstract

Acute pancreatitis (AP) is an inflammatory condition with a mild course in most patients, but 20–30% evolve to single or multiple organ dysfunction and pancreatic/peripancreatic necrosis, with potentially infected collections. In the first weeks of disease, a systemic inflammatory syndrome (SIRS) dominates the clinical setting, and early management decisions in this precocious phase can change the course of the disease. Imaging is crucial in the diagnosis, and since the adoption of the revised Atlanta classification, four different types of pancreatic/peripancreatic collections have been defined. The management of the complicated forms of AP has been defined by several treatment guidelines, and the main indication for intervention is local infection, preferably in walled-off necrosis. Open surgery necrosectomy is associated with a very high rate of morbimortality, giving a place to different multidisciplinary methodologies, emphasizing drainage and necrosectomy techniques in a “step-up” approach starting from mini-invasive endoscopic drainage and moving, if needed, to progressively more invasive techniques, including interventional radiology and mini-invasive surgery. With the advent of several new technologies in the specialties involved, the complicated AP cases which need drainage and necrosectomy benefit from a new era of multidisciplinary cooperation, permitting higher efficacy with lower levels of morbimortality and reducing hospital stay and costs.

## 1. Introduction

Acute pancreatitis (AP) is a term meaning an acute inflammatory course of the pancreas with a complete pathogenesis yet to be completed elucidated. Its definition, regardless of the aetiology, is based on the fulfilment of “two out of three” of the following criteria: Clinical (upper abdominal pain), laboratory (serum amylase or lipase >3 upper limit of normal) and imaging (computed tomography, magnetic resonance, ultrasonography) [1].

In a great majority of cases, AP is a self-limiting illness with appropriately supportive treatment. In fact, most patients with AP recover within a week, whereas patients with severe forms have a high risk of multi-organ failure [2]. These extreme forms of AP develop in 20% of patients, with a historical mortality risk of as high as 30% [3].

There have been many clinical scoring systems proposed to predict severity in the early phase of AP (Ranson [4], Modified Glasgow [5], Systemic Inflammatory Response Syndrome (SIRS) [6], and Acute Physiology, Age, Chronic Health Evaluation II (APACHE II) [7]), together with imaging-guided severity indexes (Balthazar [8], computed tomography severity index [9]), or even single laboratory parameters such as C-reactive protein, procalcitonin or blood urea nitrogen. Two recent systematic reviews confirmed the APACHE II and simple bedside index as the most accurate severity prediction scores [10,11]. In clinical practice, however, these scoring systems are irrelevant because of their complexity and low feasibility, and the decisions are based on real-time measurements [12]. 

During the first weeks of AP, there is a release of inflammatory mediators, initiating a systemic inflammatory response syndrome (SIRS) that may induce single or multiorgan failure with or without concomitant necrosis, infection, and possibly death [6]. This response can be similar, or even identical, to a clinical one arising from infection, and it was described for the first time as an inflammatory process, independent of its cause [13]. Its manifestations include, but are not limited to, more than one of the following: Temperature >38 °C or <36 °C; tachycardia >90 bpm; tachypnea > 20/min or pCO_2_ < 32 mmHg; and white blood cell count > 12000/cu mm (cubic millimeter) or <4000/cu mm; or >10% immature (band) forms [14].

Imaging is certainly very useful for the diagnosis of AP, but it is not an absolute requirement for it, as in cases of prolonged period between clinical complaints and presentation, patients with low level of consciousness, or clinical situations with a difficult differential diagnosis with other acute abdomen scenarios.

Since the adoption of the revised Atlanta classification in 2012, AP has been defined in three grades of severity: Mild, moderately severe and severe. Of these three grades, it is the severe form of AP that presents persistent organ failure for more than 48 hours despite the presence or the absence of local complications [6].

The local complications of AP have been classified in four types of collections based on radiologic and pathologic features, independently of the clinical prediction of gravity. Any of these types of fluid collections may be sterile or infected. This determinant-based classification defines two types of collections in the setting of interstitial edematous pancreatitis (acute peripancreatic fluid collections and pancreatic pseudocysts) and two other types of collections in necrotizing pancreatitis (acute necrotic collections before demarcation and walled-off necrosis (WON) [15]. This last type of collections characteristically has encapsulating walls that are developed four or more weeks after the onset of AP according the revised Atlanta classification. Nevertheless, a multicenter study demonstrated that 43% of demarcated collections already developed within the first three weeks after onset of necrotizing pancreatitis [16].

## 2. Management of Complicated Acute Pancreatitis

Since it is one of the most common gastrointestinal disorders that requires hospitalization, AP annually leads to huge inpatient costs, requiring evidence-based treatment guidelines provided by the pancreatic community: The International Association of Pancreatology (IAP) and the American Pancreatic Association (APA) [3,17]. Indeed, a systematic review of guidelines for AP emphasized the need for a high-quality update to influence several important aspects of the medical and surgical management of AP [18].

There are two overlapping phases of AP (early and late), the first one being considered in the first two weeks of the disease onset. Crucial early management decisions can change the course of AP, validating recent guidelines addressing the first 48–72 hours of admission [19]. These guidelines aim at providing evidence-based recommendations for the treatment of AP and consider both the revised Atlanta classification [6] and the most recent consensus conference on interventions for necrotizing pancreatitis [20].

One of the most cited guidelines in literature was released in 2013 by the IAP/APA working group [1], and it incorporated 12 main topics, 38 clinical questions, and their answers. 

Recently, other evidence-based international consensus statements on the management of severe AP have appeared, such as the World Society of Emergency Surgery guidelines, published in 2019. Some of these statements obtained a strong level of evidence, namely most aspects of the management in the Intensive Care Unit (ICU), opposite to others showing a quite weak evidence, such as surgical strategies that still require further studies [21].

The indications to intervene in necrotizing pancreatitis are local infections, preferably WON. Even without a documented infection, clinical deterioration for several weeks after onset justifies intervention. Almost 50% of patients operated due to persisting organ failure without signs of infected necrosis have an unexpectedly positive bacterial culture in the operative specimen [22]. In sterile necrotizing pancreatitis, other less frequent indications are clinical situations such as abdominal compartment syndrome, acute bleeding, bowel ischemia, or any type of obstruction due to mass effect [22]. Another late complication of necrotizing pancreatitis, the “disconnected duct syndrome,” may be found in up to 40% of these patients, half of them requiring an intervention due to symptoms related to a sustained main pancreatic duct injury [23].

**Figure 1:**
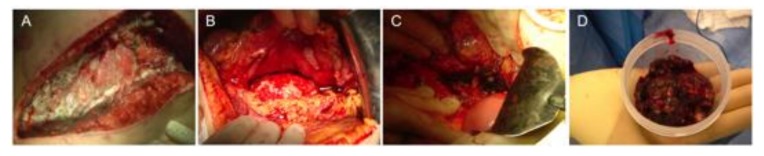
Open necrosectomy (sequential steps). (**A**) Transverse laparotomy, (**B**) retrogastric approach, (**C**) major artery skeletonized, and (**D)** necrosectomy specimen.

**The surgical “step-down” approach:** Classically, the standard of care for infected pancreatic necrosis was, until recently, surgical debridement unless patients were too ill to undergo surgical intervention [6]. Accordingly, this “step down” approach adopted open necrosectomy to play a primary therapeutic role, with less invasive methods used for residual or subsequent collections (Figure 1). This concept began to be challenged by anecdotal reports of better outcomes with less aggressive approaches, recognizing that open necrosectomy often makes patients sicker, and outcomes may be improved by simply delaying it [24]. Moreover, the understanding that most patients with sterile pancreatic necrosis and some with infected necrosis could be successfully managed without necrosectomy has influenced the change of the paradigm [25]. 

Nowadays, it is accepted that postponing surgical interventions for more than four weeks after the onset of AP results in lower mortality rates. A recent systematic review and meta-analysis compared timing of operative interventions in three different cut-offs (72 h, 12 days and 30 days) and concluded that late surgery had a clear survival benefit in all of them [26]. 

**Figure 2:**
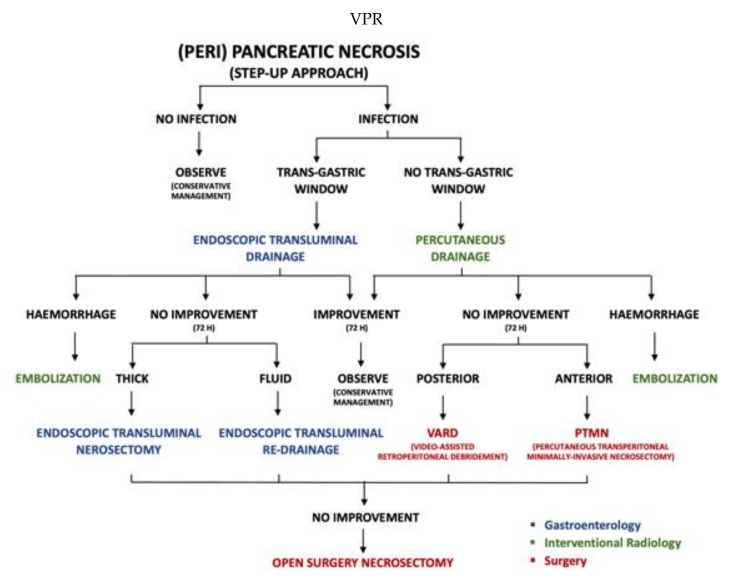
Pathways of management of complicated acute pancreatitis. Types of interventions are differentiated by colors: Surgery in red, gastroenterology in blue, interventional radiology in green.

**The multidisciplinary “step-up” approach:** As an alternative to open necrosectomy, less invasive techniques have progressively demonstrated better results, namely percutaneous drainage [27], endoscopic transgastric drainage [28] and retroperitoneal, minimally invasive necrosectomy [29]. These techniques might be used in the so-called “step up” approach, aiming at controlling the source of the infection instead of trying to completely remove the infected necrotic tissue, relying on less invasive approaches in the first step of treatment and progressively climbing up to an open necrosectomy as a last option [30]. A flowchart reporting the pathways of management of acute pancreatitis is depicted in Figure 2.

The primary step is the drainage of the infected fluid (endoscopically or percutaneously), which may defer or even avoid surgical necrosectomy. If there is no clinical improvement, the next step is a video-assisted retroperitoneal debridement [31].

A multicenter randomized trial from the Dutch Pancreatitis Study Group (the PANTER—Patients with Acute Necrotizing Pancreatitis—trial) demonstrated how the “step-up” approach decreased mortality, major complications and costs, when compared to primary necrosectomy among patients with necrotizing pancreatitis and infected necrotic tissue [32].

In the PANTER study, 35% of patients were successfully treated with percutaneous drainage alone, without subsequent debridement. More than 95% of patients with infected necrosis can be drained percutaneously, but a promising alternative is undoubtedly the NOTES (natural orifice transluminal endoscopic surgery) route for drainage and/or necrosectomy. The endoscopic technique of cystogastrostomy has evolved in the last decade to eventually become the first choice as minimally invasive necrosectomy in most cases, when compared to minimally invasive intervention; two pilot trials, the Pancreatitis Endoscopic Transgastric versus Primary Necrosectomy in Patients with Infected Pancreatic Necrosis (PENGUIN) [33] and the Transluminal Endoscopic Step-Up approach versus Minimally Invasive Surgical Step-Up approach (TENSION) [34] showed at least an equivalence between the two procedures.

Transgastric EUS (endo-ultrasonography) guided drainage is the treatment of choice for late and persistent pancreatic collections, except, according with some authors, some lateral and retrocolic-ones [35]. However, in the case of failure of endoscopic or catheter drainage, the methodology for necrosectomy is unclear.

**Figure 3:**
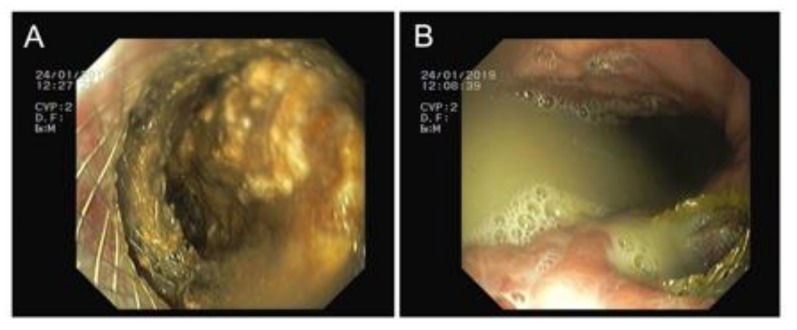
Transmural route. (**A**) Necrosectomy through the prosthesis and (**B**) purulent drainage to the gastric lumen.

**Figure 4:**
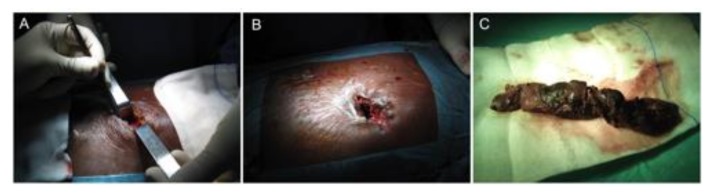
Transperitoneal route. (**A**) Minimally invasive skin incision, (**B**) lesser sac entrance, and (**C**) complete necrosectomy specimen.

To achieve a minimally invasive necrosectomy, several approaches have been described that utilize endoscopic approaches, image-guided techniques and small incision open surgeries and are performed by gastroenterologists, interventional radiologists or surgeons. This has resulted in a confusing array of terms describing these procedures, demanding a common terminology. A classification of invasive procedures has proposed that describes visualization, route and purpose (the VRP Classification) [36], establishing different competences for each involved specialty: i) Per-os transpapillary route (gastroenterology); ii) per-os transmural route (gastroenterology) (Figure 3); iii) percutaneous retroperitoneal route (surgeon/interventional radiologist); iv) percutaneous transperitoneal route (surgeon) (Figure 4); and percutaneous transmural route (surgeon/interventional radiologist).

More recently (2018), The European Society of Gastrointestinal Endoscopy (ESGE) released several evidence-based multidisciplinary guidelines [37], such as: (i) The fine needle aspiration of (peri)pancreatic collections only if infection is suspected; (ii) EUS—guided access should be preferred to conventional transmural drainage in a first approach; and (iii) simultaneous drainage (endoscopic, transmural, and percutaneous) in WON extending to pelvic paracolic gutters.

Any variety of minimally invasive necrosectomy is dependent of the proper control of hemorrhage to be successful. Moreover, pancreatitis itself can cause both thrombotic and hemorrhagic complications through the formation of a fluid collection, with a significant morbidity and mortality. These complications occur in between 1% and 23% of pancreatitis cases, with venous being significantly more common than arterial ones [38].

Despite their rarity, it is important to identify and treat arterial complications as early as possible, as they are associated with a mortality of 34–52% [39]. Arterial wall disruption can occur due to the exposure to the free lipolytic and proteolytic enzymes, leading to pseudo-aneurysm formation or spontaneous arterial rupture.

Bleeding can also occur from the disruption of the wall of a pseudocyst or WON. The presence of endoscopically or surgically inserted drains can also directly traumatize vessels. They can perpetuate local inflammation and diminish arterial wall integrity.

Any form of necrosectomy can disrupt arterial viability and cause multi-organ failure, necrosis, anticoagulation and underlying vasculitis [40].

**Figure 5:**
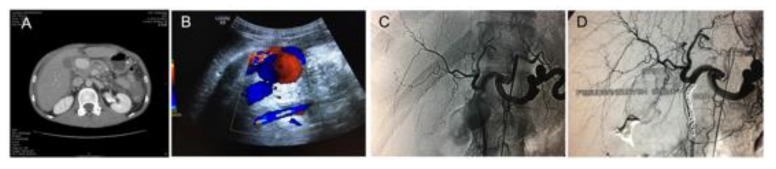
Diagnosis and treatment of a pseudo-aneurysm of the gastroduodenal artery in a patient with severe acute pancreatitis. (**A**) Abdominal angio-CT revealing a large contrast-enhanced mass, (**B**) characteristic swirl flow in abdominal ultrasound-Yin-Yang sign, (**C**) digital subtraction angiography (DSA) showing the large pseudoaneurysm projected over the gastroduodenal artery, and (**D**) DSA showing the post trans-arterial embolization of the gastroduodenal artery with multiple coils.

Symptomatic pseudo-aneurysms present abdominal pain as the main complaint in 29.5% of patients, followed by bleeding into the gastrointestinal tract in 26.5%. They can occur in the splenic artery (35–50%), gastroduodenal artery (30%, Figure 5), or pancreaticoduodenal arcades (20–25%) [41]. Imaging diagnosis is based in the Angio-Computed Tomography (CT) and ultra-sonography Doppler imaging (Figure 5). Treatment is performed by trans-arterial embolization through the placement of coils or other embolic agents in the proximal and distal feeding vessel (the “frontdoor” and “backdoor” technique) to isolate inflow and prevent collateral back filling (Figure 5).

Interventional radiology also has a very important rescuing contribution to the approach of complicated cases, both in the imaging diagnosis and treatment of hemorrhagic situations after necrosectomy procedures by endoscopic or previous surgery [42].

**Figure 6:**
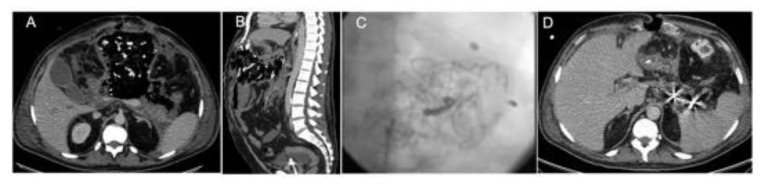
Splenic artery trans-arterial embolization by the placement of coils in a patient with acute pancreatitis patient submitted to an “open abdomen” tamponade for hemorrhage control. (**A**) Axial CT view, (**B**) sagittal CT view, (**C**) angiographic view post-embolization, and (**D**) axial CT control after embolization, with metal artifacts from the coils placed in the splenic artery.

With the rise of minimally invasive techniques (angiographic, percutaneous, or endoscopic), the role of surgery has become restricted. Still, despite constant technological advances in other areas of intervention, open surgery continues to play a rescue role in hemorrhage control where other methods are not available or technically achievable. In Figure 6, we present the case of a patient transferred to our center with an “open abdomen” with tamponade to control an overwhelming hemorrhage caused by acute pancreatitis. He had been submitted to more than one open necrosectomy before being transferred to our center. The first approach was by intervention radiology with the trans-arterial embolization of the splenic artery, followed by open surgery with abdominal closure.

Conventional surgery still has a role in the treatment of complicate AP when percutaneous or endoscopic strategies fail to improve the patient, namely: (i) Abdominal compartment syndrome, requiring surgical decompression, if other conservative measures are insufficient [43,44]; (ii) cholecystectomy: Only in cases where a deferred strategy that enables the resolution of fluid collections is not possible [45]; and (iii) mechanical bowel obstruction [46].

## 3. Conclusions

Complicated acute pancreatitis remains a devastating disease that is associated with mortality up to 30% and enormous and rising inpatient costs [17]. Early triage and referral to high-volume centers with defined protocols and multidisciplinary approaches are ways to drop of the global mortality described in literature in the last few decades. 

Interventions for necrotizing pancreatitis have evolved considerably over recent years, with a minimally invasive step-up approach now preferred. The best methodology, according to the literature, is endoscopic and/or percutaneous catheter drainage followed, if needed, by endoscopic or minimally invasive surgical necrosectomy. 

A highly differentiated team with a special interest in necrotizing pancreatitis is mandatory to succeed with the best outcomes, clinically and financially. Such a team must integrate dedicated pancreatic surgeons, gastroenterologists, and intervention radiologists beyond intensivists in appropriate intensive care unit facilities.
